# The Therapeutic Potential of Bombyx Batryticatus for Chronic Atrophic Gastritis Precancerous Lesions via the PI3K/AKT/mTOR Pathway Based on Network Pharmacology of Blood-Entering Components

**DOI:** 10.3390/ph18060791

**Published:** 2025-05-25

**Authors:** Xiaojie Wang, Miaomiao Chang, Kun Feng, Qingyue Wang, Bowen Li, Weijuan Gao

**Affiliations:** Hebei Key Laboratory of Chinese Medicine Research on Cardio-Cerebrovascular Disease, Hebei University of Chinese Medicine, Shijiazhuang 050200, China; xjwang1066@163.com (X.W.); changmiao178@163.com (M.C.); 18838225083@163.com (K.F.); 15032220688@163.com (Q.W.)

**Keywords:** chronic atrophic gastritis precancerous lesions, Bombyx Batryticatus; UPLC-QE-Orbitrap-MS/MS; network pharmacology; PI3K/AKT/mTOR pathway, Bombyx Batryticatus, UPLC-QE-Orbitrap-MS/MS, network pharmacology, PI3K/AKT/mTOR pathway

## Abstract

**Background:** Chronic atrophic gastritis precancerous lesions (PL-CAG) are characterized by the atrophy of gastric mucosal glands, often accompanied by intestinal metaplasia or dysplasia. Timely intervention and treatment can effectively reverse its malignant progression and prevent the onset of gastric cancer. Bombyx Batryticatus (BB) exhibits a range of pharmacological effects, including anticoagulation, antiepileptic properties, anticancer activity, and antibacterial effects. However, the pharmacological basis and mechanisms underlying BB’s efficacy in treating PL-CAG remain unclear. **Methods:** A three-factor modeling approach was implemented to develop a rat PL-CAG model, while the MNNG-induced PLGC (precancerous lesions of gastric cancer) cell model was served as a cell PL-CAG model. UPLC-QE-Orbitrap-MS/MS (Ultra performance liquid chromatography-quadrupole-electrostatic field orbital trap high-resolution mass spectrometry) was utilized to perform an in-depth analysis of the components in the plasma extract of BB. Leveraging network pharmacology, molecular docking analyses, and experimental validation, we initially elucidated the potential mechanisms through which BB mediates its therapeutic effects on PL-CAG at both in vivo and in vitro levels. **Results:** Prototype compounds of 42 blood-entering components were identified by UPLC-QE-Orbitrap-MS/MS analysis. Network pharmacology analysis and molecular docking studies indicate that the core targets are primarily enriched in the PI3K-Akt signaling pathway, and the key components, including Nepitrin, Quercetin 3-*O*-neohesperidoside, Rutin, and others, exhibited stable docking conformations with the first eleven pivotal targets. Both in vivo and in vitro experiments validated that BB may effectively treat PL-CAG via modulation of the PI3K-Akt signaling pathway. **Conclusions:** The therapeutic efficacy of BB in the management of PL-CAG may be achieved through the synergistic interaction of multiple components and targets, which may be more closely related to the inhibition of the PI3K/AKT signaling pathway. This approach will establish a solid experimental foundation and provide essential data for the clinical application of BB in treating PL-CAG, while also facilitating further research initiatives.

## 1. Introduction

Gastric cancer represents a significant public health burden in China, contributing to 44.0% of global cases and 48.6% of related deaths in 2020 [1]; the escalating incidence and mortality rates underscore the urgent need for effective interventions. Chronic atrophic gastritis (CAG), a chronic digestive disorder marked by gastric mucosal gland degeneration, is a well-established precursor to gastric cancer when accompanied by intestinal metaplasia and dysplasia [2]. Early detection and intervention are critical to mitigating disease progression. However, the nonspecific clinical presentation of CAG has hindered the development of effective pharmacological therapies in Western medicine. Traditional Chinese medicine (TCM) offers a promising alternative, promoting gastric mucosal regeneration and potentially reversing atrophy, thereby reducing cancer risk with minimal adverse effects.

Bombyx Batryticatus (BB), a traditional medicine in Chinese pharmacopeia, is classified as a product of ‘blood, flesh, and emotions’. First documented in the *Shennong Bencao Jing*, BB is also known as Bai Jiang Can or Tian Chong. BB is derived from the dried larvae of *Bombyx mori* infected by the fungus *Beauveria bassiana*, undergoing sclerotization during processing [3]. According to TCM theory, BB exhibits a neutral nature, a salty flavor profile, and therapeutic effects on the liver, spleen, and stomach meridians. BB demonstrates diverse pharmacological activities, including wind-calming, convulsion-mitigating, pain-alleviating, phlegm-resolving, and nodule-dispersing properties [4]. Clinical studies have validated BB’s efficacy in treating diverse conditions, such as lymphatic tuberculosis, lung cancer, nasopharyngeal carcinoma, bladder cancer, brain tumors, malignant lymphoma, and breast cancer.

Rooted in systems biology, network pharmacology employs multi-dimensional networks to map the complex interactions between TCM components, molecular targets, and biological pathways, offering a holistic understanding of therapeutic mechanisms. This approach aligns with the holistic philosophy of TCM, providing a systems-level perspective on pharmacological mechanisms [5].

UPLC-QE-Orbitrap-MS/MS provides exceptional resolution, rapid scanning, and precise structural characterization. This technology enables simultaneous, high-throughput identification and quantification of multiple components without requiring reference standards. As a result, UPLC-QE-Orbitrap-MS/MS has been widely adopted for rapid screening and qualitative analysis of both single-ingredient and compound TCM formulations [6].

In this study, we integrated UPLC-QE-Orbitrap-MS/MS, network pharmacology, molecular docking, and experimental validation to elucidate the pharmacological mechanisms underlying BB’s anti-PL-CAG effects. This integrative approach not only establishes a robust experimental foundation for BB’s clinical application in PL-CAG management but also paves the way for future mechanistic investigations.

## 2. Results

### 2.1. BB Can Mitigate the Pathological Damage in PL-CAG Rats and Enhance Their Gastric Function

**Figure 1:**
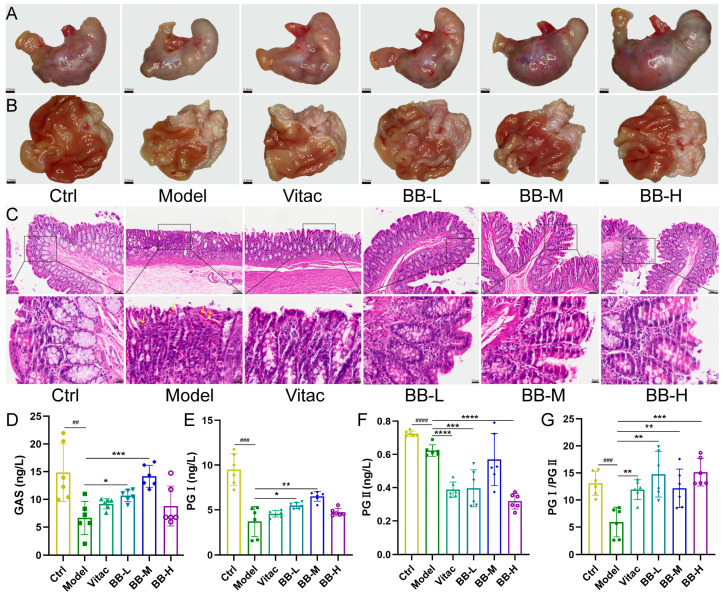
Effects of BB on CAG rats. (**A**) Macroscopic examination of the stomachs of different groups of rats. (**B**) Macroscopic examination of the gastric mucosa of different groups of rats. (**C**) Typical representative sections of HE staining of gastric histopathology in rats (the yellow arrow indicates capillary congestion). Effect of BB on serum levels of GAS (**D**), PG I (**E**), PG II (**F**), PG I /PG II (**G**). Results were represented as mean ± SD. *n* = 6. Compared to control, ^##^ *p* < 0.01, ^###^ *p* < 0.001, ^####^ *p* < 0.0001. Compared to model, * *p* < 0.05, ** *p* < 0.01, *** *p* < 0.001, **** *p* < 0.0001.

Compared with the Ctrl group, the Model group showed a decreased stomach volume and a paler color. Visual inspection indicated a thinner mucosal layer with fewer folds. HE staining further demonstrated an irregular arrangement of epithelial cells, atrophy, reduced gland density, vascular congestion, inflammatory cell infiltration, and Dys. In contrast, the treatment groups exhibited an increased stomach volume and thicker mucosal layers with more prominent folds upon visual inspection. HE staining revealed marked improvements in the pathological changes of the gastric mucosa (Figure 1A–C). Compared with the Model group, the levels of gastrin (GAS) and pepsinogen I (PG I) and the ratio of PG I/PG II were significantly decreased in the Model group (*p* < 0.01). In contrast, the treatment groups showed marked increases in the levels of GAS and PG I and the ratio of PG I/PG II (*p* < 0.05, *p* < 0.01) (Figure 1D–G).

### 2.2. BB Mitigates the MNNG-Induced Upregulation of Inflammatory Factors in the PL-CAG

**Figure 2:**
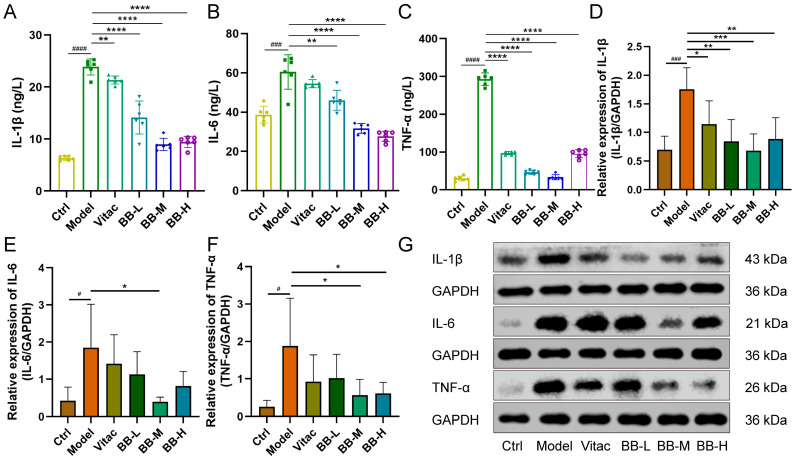
Expression of inflammatory factors in rats. Effect of BB on serum levels of IL-1β (**A**), IL-6 (**B**), and TNF-α (**C**). (**D**–**G**) Representative Western blot images and bar graphs of the relative expressions of IL-1β, IL-6, and TNF-α in each group. Results were represented as mean ± SD. *n* = 6. Compared to control, ^#^ *p* < 0.05, ^###^ *p* < 0.001, ^####^ *p* < 0.0001. Compared to model, * *p* < 0.05, ** *p* < 0.01, *** *p* < 0.001, **** *p* < 0.0001.

Compared with the Ctrl group, the levels of IL-1β, IL-6, and TNF-α in the Model group were significantly increased (*p* < 0.01). Compared with the Model group, the levels of IL-1β, IL-6, and TNF-α in the treatment groups were significantly decreased (*p* < 0.01), especially in the BB-M group (Figure 2).

### 2.3. BB-Containing Serum Inhibits the Proliferation, Migration, and Invasion of PLGC Cells

**Figure 3:**
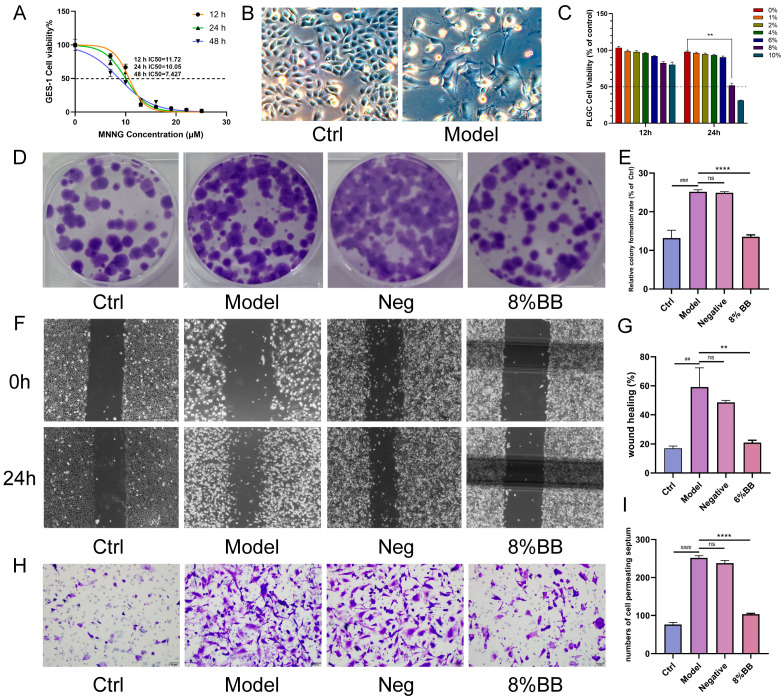
Impact of BB on the proliferation, migration, and invasion of PLGC cells in vitro. (**A**) The IC50 value of MNNG was determined by CCK-8 assay for PLGC cells. (**B**) Morphological observation of normal gastric mucosal epithelial cells (GES-1) and MNNG-induced PLGC cells. Scale bar: 200 μm. (**C**) The effect of BB serum-containing on the viability of PLGC cells was assessed by CCK-8 assay. (**D**,**E**) Colony formation assay of PLGC cells that were treated with the BB drug serum. Representative images and quantification of cell colonies are shown. (**F**,**G**) The effect of BB drug serum on the migration of PLGC cells was evaluated by scratch healing assays. Representative images and quantification of the wound healing rate are shown. Scale bar: 500 μm. (**H**,**I**) The effect of BB drug serum on the invasion of PLGC cells was evaluated by Transwell assays. Representative images and quantification of invading PLGC cells are shown. Scale bar: 50 μm. Data are presented as mean ± SD. *n* = 3. Compared to control, ^##^ *p* < 0.01, ^###^ *p* < 0.001, ^####^ *p* < 0.0001. Compared to model, ** *p* < 0.01, **** *p* < 0.0001.

The optimal time and concentration for MNNG-induced PLGC cells were detected through CCK-8 experiments (Figure 3A). The successful induction of PLGC cells was observed under a microscope (Figure 3B). CCK-8 experiments revealed that serum containing 8% BB markedly inhibited the growth of PLGC cells after 24 h of treatment (Figure 3C). Colony formation assays further confirmed that the BB-containing serum significantly suppressed the proliferation of PLGC cells (Figure 3D–E). Additionally, wound healing and Transwell migration assays demonstrated that the BB-containing serum substantially diminished the migratory capacity of PLGC cells (Figure 3F–I).

### 2.4. Components of BB Prototype That Can Enter the Bloodstream

**Figure 4:**
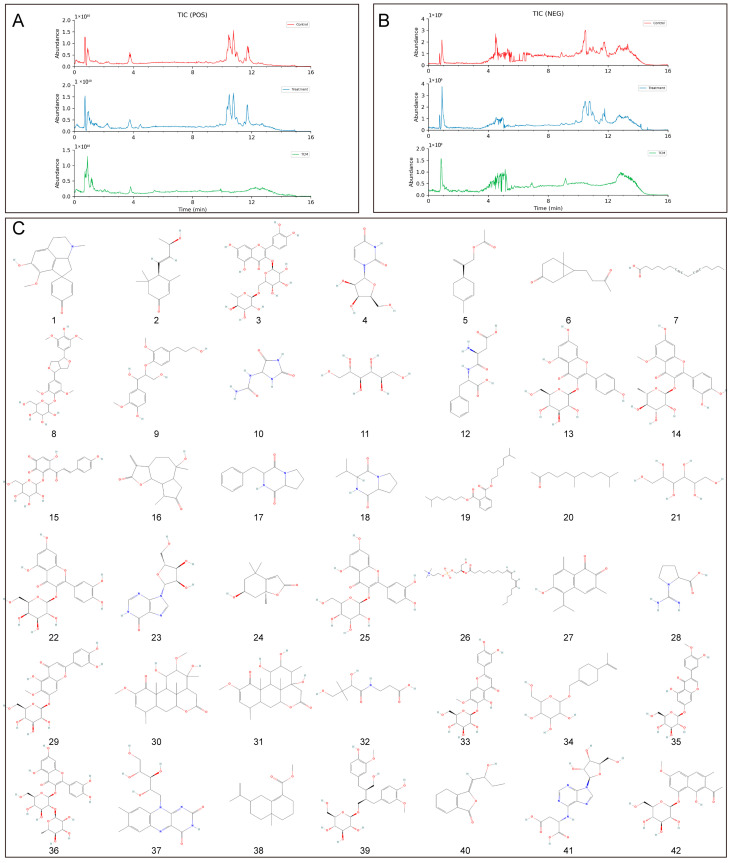
BB chromatogram and prototype compounds of blood-entering components. (**A**) Total ion chromatograms (TICs) recorded in the positive ion mode of BB. (**B**) TICs recorded in the negative ion mode of BB. (**C**) Structures of compounds 1–42.

After the aqueous extract of BB was absorbed into the blood by gavage in rats, a total of 52 chemical components were identified in BB-containing plasma. By comparative analysis with the chemical components in the aqueous extract, prototype compounds of 42 blood-entering components were obtained (Figure 4, Table 1).

### 2.5. Network Pharmacology Suggests That BB Alleviates PL-CAG via the PI3K/AKT Pathway

**Figure 5:**
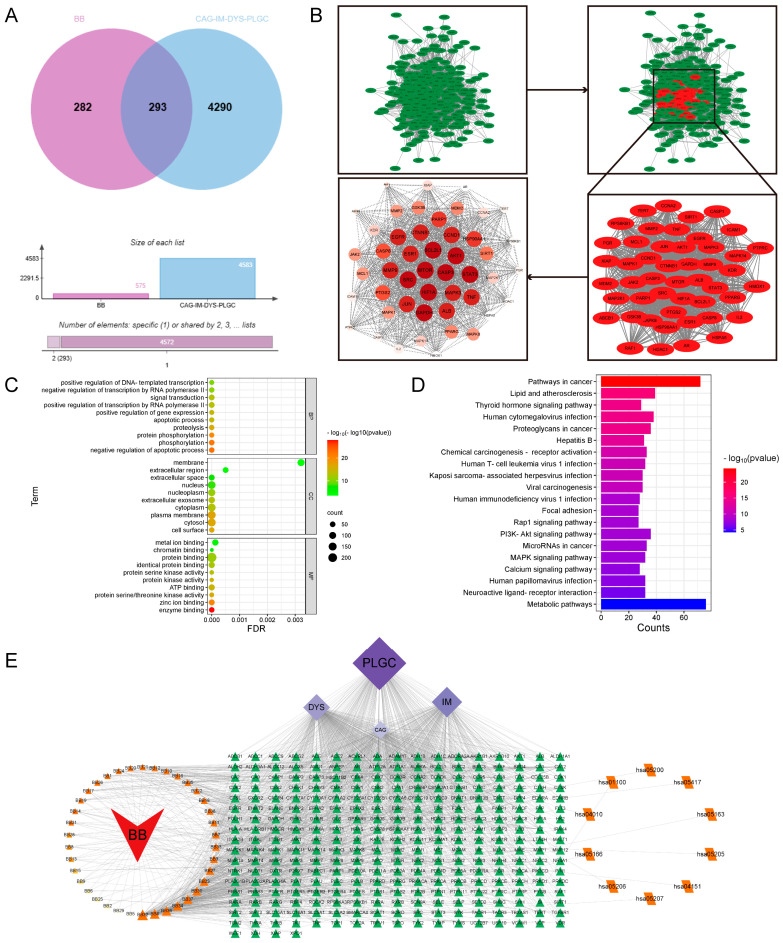
Network analysis of BB treating PL-CAG. (**A**) Venn diagram of 293 common targets of BB and CAG. (**B**) Acquirement of the 48 key targets from the 293 common targets via STRING network topological analysis. (**C**) The top 10 significantly enriched terms in biological processes, molecular functions, and cellular components. (**D**) The top 20 significantly enriched terms in KEGG pathways. (**E**) Drug–components–diseases–targets–pathway network. Illustration of the relevance among components of BB, the four stages of CAG progression (CAG, IM, DYS, PLGC), the key targets, and the top corresponding pathways. Orange nodes refer to the 42 components of BB, purple nodes refer to the four stages of CAG progression, green nodes refer to the key targets, and yellow nodes refer to the signaling pathways.

**Figure 6:**
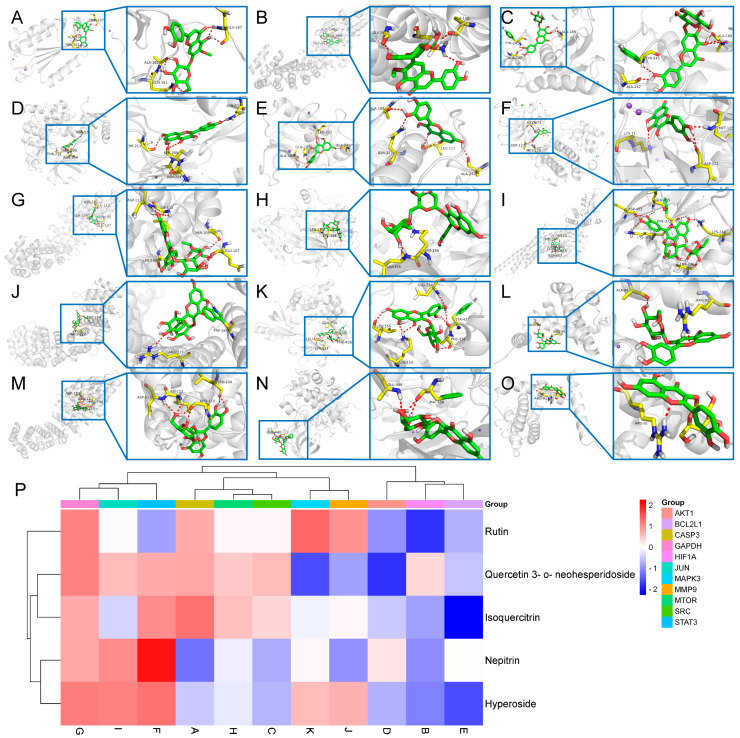
Molecular docking results. (**A**–**O**) The representative docking complex of key targets and compounds. (**P**) The heatmap of docking scores of key targets combining the top 5 active compounds in BB.

In total, 293 intersection genes were obtained by Venny mapping as potential targets for BB treatment of PL-CAG (Figure 5A). The potential targets were entered into the STRING platform, and the protein–protein interaction (PPI) network showed 291 nodes and 5649 edges. Using Cytoscape 3.9.1 software, the PPI protein network was clustered and analyzed by MCODE and sorted according to the degree value to obtain 48 core targets such as *CASP3*, *HIF1A, SRC*, *AKT1*, *BCL2L1*, *STAT3*, *GAPDH*, *MTOR*, *JUN*, *MMP9*, and *MAPK3* (Figure 5B). Gene ontology (GO) functional enrichment showed that there were 929 biological processes, mainly involving protein phosphorylation, negative regulation of apoptotic process and signal transduction. There were 104 cellular components, mainly including cytoplasm, plasma membrane, and nucleus, and 231 molecular functions, mainly involving protein binding, ATP binding, and enzyme binding (Figure 5C). Kyoto Encyclopedia of Genes and Genomes (KEGG) enrichment analysis yielded a total of 191 pathways, which are mainly involved in signaling pathways such as pathways in cancer, PI3K-Akt signaling pathway, etc. (Figure 5D). Using Cytoscape 3.9.1 software, the network of drug–components–diseases–targets–pathways was constructed, and the main active ingredients such as Nepitrin, Quercetin 3-*O*-neohesperidoside, Rutin, Isoquercitrin, and Hyperoside were screened according to the degree value (Figure 5E). The 11 core targets *CASP3*, *HIF1A*, *SRC*, *AKT1*, *BCL2L1*, *STAT3*, *GAPDH*, *MTOR*, *JUN*, *MMP9*, *and MAPK3* were combined with the main active ingredients Nepitrin, Quercetin 3-*O*-neohesperidoside, Rutin, Isoquercitrin, and Hyperoside (Figure 6A–O). The molecular docking results showed that most of the active ingredients had good binding activity to the target proteins (Figure 6P).

### 2.6. Effect of BB on the Expression of PI3K/AKT/mTOR Signaling Pathway-Related Proteins in Gastric Tissues of PL-CAG Rats

**Figure 7:**
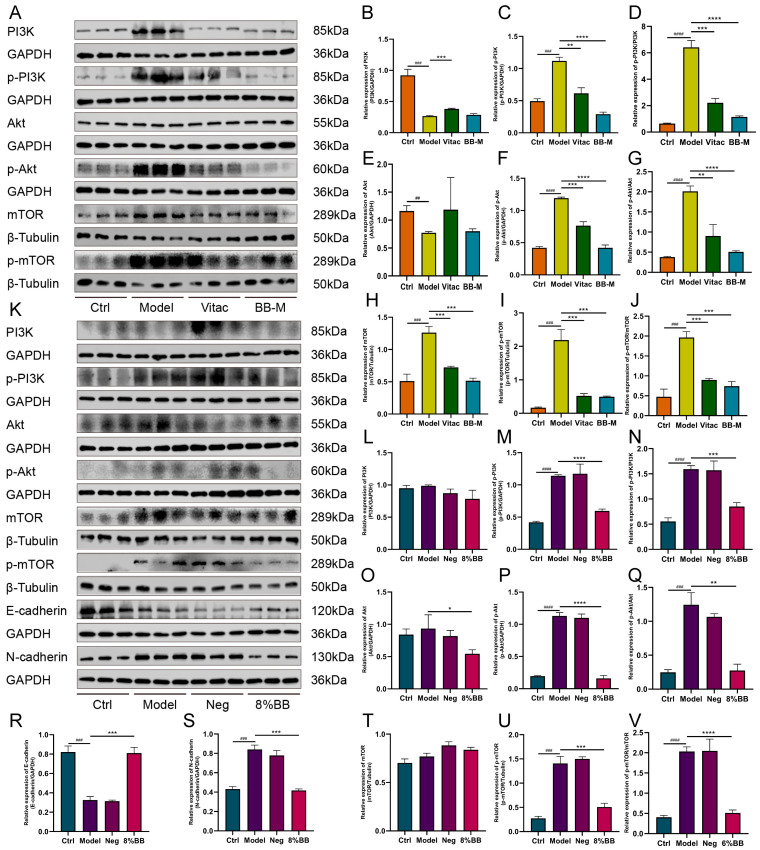
BB suppresses the activation of the PI3K/AKT/mTOR signaling pathway in vivo and in vitro. (**A**) The impact of BB on the expression of proteins associated with the PI3K-AKT signaling pathway in vivo. (**B**–**J**) Representative Western blot images and bar graphs of the relative expressions of PI3K, p-PI3K, Akt, p-Akt, mTOR, and p-mTOR in rats. (**K**) The impact of BB on the expression of proteins associated with the PI3K-AKT signaling pathway in vitro. (**H**–**V**) Representative Western blot images and bar graphs of the relative expressions of PI3K, p-PI3K, Akt, p-Akt, mTOR, p-mTOR, E-cadherin, and N-cadherin in PLGC cell. Results were represented as mean ± SD. n = 6. Compared to control, ^##^ *p* < 0.01, ^###^ *p* < 0.001, ^####^ *p* < 0.0001. Compared to model, * *p* < 0.05, ** *p* < 0.01, *** *p* < 0.001, **** *p* < 0.0001.

Select the BB-M group, which exhibits more pronounced pharmacological effects, for pathway mechanism research. Compared with the Ctrl group, the expression of p-PI3K, p-Akt, and p-mTOR protein levels in the Model group were significantly increased (*p* < 0.01, *p* < 0.001). The expression of p-PI3K/PI3K, p-Akt/Akt, and p-mTOR/mTOR protein levels were significantly decreased in the Vitac group and BB-M groups compared with the Model group (*p* < 0.01, *p* < 0.001). The BB-M group was superior to the Vitac group (Figure 7A–J).

### 2.7. Effects of BB-Containing Serum on the Expression of Epithelial–Mesenchymal Transition Proteins E-Cadherin and N-Cadherin and Proteins Related to PI3K/AKT/mTOR Signaling Pathway in PLGC Cells

Compared with the Ctrl group, E-cadherin protein expression was decreased and N-cadherin, p-PI3K, p-Akt, and p-mTOR protein expression was increased in the Model group (*p* < 0.001). Compared with the Model group, E-cadherin protein expression was increased in the BB-containing serum group, and N-cadherin, p-PI3K, p-Akt, and p-mTOR protein expression was decreased in the blank serum group (*p* < 0.05, *p* < 0.01). However, there was no significant difference between the blank serum groups and the Model group (*p* > 0.05) (Figure 7K–V).

## 3. Discussion

First proposed by Correa in 1975, the widely accepted multi-stage gastric carcinogenesis model delineates a progression sequence from normal gastric mucosa to chronic superficial gastritis, precancerous lesions, and ultimately invasive gastric cancer (GC) [7]. The World Health Organization classifies CAG presenting with intestinal metaplasia (IM) and dysplasia (Dys) as gastric precancerous lesions (PLGC). In this work, we collectively term these pathological states—CAG with or without IM/Dys—as precancerous lesions of chronic atrophic gastritis (PL-CAG) to unify mechanistic investigations.

Current therapeutic strategies in modern medicine lack an effective oral pharmacotherapy specifically targeting PL-CAG. Clinical management predominantly relies on pharmacological interventions including proton pump inhibitors, antibiotics, acid suppressants, and gastric mucosal protectants. While these agents demonstrate efficacy in mitigating mucosal inflammation and relieving abdominal pain symptoms, prolonged administration may induce adverse effects such as hepatorenal toxicity, multidrug resistance development, and osteoporosis risk [8]. In contrast, TCM presents a multi-target therapeutic approach with enhanced safety profiles [9], offering distinct advantages for PL-CAG management.

The Chinese Pharmacopoeia documents BB’s pharmacological properties as pungent–salty flavor with thermodynamic neutrality, exhibiting meridian tropism towards hepatic, splenic, and gastric systems. BB has the effect of soothing hepatic wind and relieving spasm, expelling wind and relieving pain, eliminating phlegm, and dispersing knots. It is often used as a small compound imperial medicine [10]. Modern research on BB suggests that it comprises a diverse array of chemical compounds and demonstrates a spectrum of pharmacological activities.

In the clinical treatment of CAG, especially PLGC with traditional Chinese medicine, BB is often used as an important component of Chinese herbal compound. Professor LiuXiang Huang, a distinguished expert in traditional Chinese medicine, posits that CAG with atypical hyperplasia is predominantly attributed to phlegm and blood stasis, as well as intense heat and toxicity. His clinical protocol advocates therapeutic formula modification through BB integration to address these pathophysiological characteristics [11]. Grand Master Diangui Li posits that the primary pathogenesis of CAG is the accumulation of turbid toxins. His therapeutic strategy emphasizes BB-mediated detoxification and meridian regulation, particularly effective in intestinal metaplasia cases with turbid toxin manifestations [12]. Shuwen Shen, a distinguished Chinese physician, posits that in the management of PLGC, particularly among patients with gastrointestinal polyps, the combination of BB and wu mei can be employed to effectively dissolve phlegm and disperse nodules [13].

Therefore, this study initially validated the pharmacological efficacy of BB in treating CAG-PLGC through both in vivo and in vitro approaches.

This investigation established a comprehensive evaluation system that encompasses morphological, histological, and molecular biomarkers to assess PL-CAG progression and therapeutic response, including gastric mucosal morphology, histopathological features, functional biomarkers, and inflammatory mediators. Gastric mucosal metaplasia often presents with a colorectal type, which is closely linked to the development of gastric cancer. The diagnostic criterion for this condition is the presence of goblet cells within the gastric mucosa. Gastric pepsinogen (PG) is secreted by chief cells, and its serum levels decrease in the context of PL-CAG; lower PG I/PG II levels are closely associated with the occurrence of gastric cancer [14]. Gastrin (GAS), a polypeptide hormone, is produced by G cells situated in the gastric and duodenal mucosa adjacent to the stomach. Its primary role involves stimulating the secretion of gastric acid and pepsinogen while also supporting the functionality of parietal cells [15]. Interleukin-1β (IL-1β) functions not only as a key pro-inflammatory mediator but also as a powerful inhibitor of gastric acid secretion, leading to a substantial decrease in both gastric juice volume and total acidity, which may result in delayed gastric emptying [16]. Additionally, IL-1β promotes the synthesis of cyclooxygenase-2 (COX-2) [17], thereby exacerbating damage to the gastric mucosa. Interleukin-1β has the potential to induce tumorigenesis through the prolonged activation of oncogenes [18]. Tumor Necrosis Factor-alpha (TNF-α) has the potential to induce cellular immortality by modulating telomerase activity [19]. It can also cause DNA damage and promote genetic mutations [20]. Interleukin-6 (IL-6) is a widely recognized biomarker of inflammation. During an inflammatory response, the concentration of IL-6 in serum increases rapidly. Following the resolution of inflammation, its levels promptly return to baseline [21]. Our study demonstrates that, compared with the normal control group, rats in the Model group exhibited weight loss. Gross observation revealed that the gastric mucosa appeared grayish-white with a granular texture and flattened folds. HE staining results indicated mucosal epithelial hyperemia, disordered arrangement of lamina propria glands, occasional goblet cells, and extensive infiltration of inflammatory cells into the submucosa. ELISA results showed decreased serum levels of GAS and PGI/PGII, along with elevated expression levels of pro-inflammatory factors. Western blot analysis further confirmed increased expression of pro-inflammatory factors in the tissue. And after BB treatment, these pathological conditions have been significantly improved, with the improvement effect exceeding that observed in the positive control group. These findings collectively confirm the effectiveness of BB in treating rats with PL-CAG. Certainly, in the present study, we primarily focused on the changes in levels of IL-1β, TNF-α, and IL-6. However, we acknowledge the importance of evaluating additional inflammatory markers such as COX-2 and NF-κB in future investigations. COX-2 is an inducible enzyme involved in prostaglandin synthesis, which plays a critical role in mediating inflammatory responses. NF-κB serves as a pivotal transcription factor that regulates the expression of multiple inflammation-related genes, including IL-1β, IL-6, and TNF-α. These factors will constitute key areas of focus in our future research on the anti-inflammatory mechanisms of BB.

To elucidate the mechanism of drug action, we conducted pharmacodynamic validation at the cellular level. This study employed MNNG to treat GES-1 cells for a duration of 24 h. Subsequently, the normal culture medium was replaced, and the cells were cultured for an additional week to establish a PL-CAG cell model, hereafter referred to as the PLGC cell. Studies have shown that MNNG is widely used in the construction of gastric cancer precursor cell models. Following treatment with MNNG, the GES-1 cells exhibit morphological and growth characteristics typical of tumor cells. This study demonstrated that an 8% concentration of drug-containing serum significantly suppressed the proliferation, migration, and invasion capabilities of PLGC cells. These results suggest that the BB drug-containing serum exhibits therapeutic potential at the cellular level for treating PL-CAG.

According to the principles of serum pharmacochemistry in TCM, the pharmacological effects of these medicinal substances are genuinely manifested by their serum-translocating constituents. To further elucidate the pharmacological basis and mechanism of action of BB in treating PL-CAG, this study utilized UPLC-QE-Orbitrap-MS/MS technology to analyze and identify the key components of BB that enter systemic circulation. This analysis provides a scientific foundation for understanding the pharmacologically active constituents of BB and guiding mechanistic investigations into its role in PL-CAG treatment. This study analyzed and determined the prototype compounds of 42 BB components that enter the bloodstream. By employing network pharmacology and molecular docking techniques, we investigated the target sites and associated pathways linked to these bloodstream components in their therapeutic effects on CAG, IM, Dys, and PLGC.

Through the construction of a component–target network and PPI analysis, the key active components of BB in treating PL-CAG were identified as Nepitrin, quercetin-3-*O*-neohesperidoside, Rutin, Hyperoside, and Isoquercitrin. The core targets associated with these components include *CASP3*, *HIF1A, SRC*, *AKT1*, *BCL2L1*, *STAT3*, *GAPDH*, *MTOR*, *JUN*, *MMP9*, and *MAPK3*. GO and KEGG enrichment analyses revealed that the therapeutic effects of BB on PL-CAG are involved in cellular biological processes such as protein phosphorylation and enzyme binding, as well as pathways related to cancer progression, PI3K-Akt signaling pathway, and lipid metabolism disorders.

To further investigate the specific binding sites where BB exerts its aforementioned effects, we performed molecular docking validation. Generally, the results of molecular docking are primarily assessed based on the minimum binding free energy [22]. Through molecular docking analysis, we discovered that among the top 11 targets exhibiting stronger affinity for the main component, core effector molecules AKT1 and mTOR within the PI3K/AKT/mTOR signaling pathway were identified.

AKT is a serine/threonine protein kinase categorized into three subtypes: Akt1, Akt2, and Akt3, and it functions as a downstream effector influenced by PI3K signaling. Among these, Akt1 is ubiquitously expressed across all tissue types, including the stomach. By contrast, Akt2 and Akt3 are scarcely detected in gastric tissues. As a critical regulatory kinase, Akt1 orchestrates cell growth and survival through the phosphatidylinositol 3-kinase (PI3K)-Akt signaling pathway [23]. The activation of PI3K promotes the translocation of Akt to the cell membrane, where it is phosphorylated by PIP3, leading to its activation. The phosphorylated form of Akt, denoted as p-Akt, acts as a critical marker for evaluating the degree of Akt activation and can further activate multiple downstream effectors. Both PI3K and Akt are frequently overexpressed in various cancer cell types and play significant roles in cancer metastasis [24]. Furthermore, a substantial body of evidence has shown that the activation of the Akt signaling pathway is frequently correlated with increased tumor invasiveness in various human cancers [25].

mTOR is a serine/threonine kinase, which is a downstream effector molecule of the PI3K/AKT signaling pathway. The activation of mTOR is typically achieved through phosphorylation, a process that is regulated by various signals [26]. mTOR, as a crucial role in intracellular key signal transduction pathways, is involved in regulating cell growth, proliferation, and metabolism. Studies have shown that the abnormal activation of the mTOR signaling pathway is closely related to the occurrence and development of tumors in various cancers. For instance, in pancreatic cancer, the inhibition of the mTOR signaling pathway can effectively promote the phenotypic transformation of cancer stem cells, thereby inhibiting their metastatic ability [27]. In renal cell carcinoma, overexpressed exogenous proteins can enhance epithelial–mesenchymal transition (EMT) through the mTOR signaling pathway, thereby promoting the migration and invasion capabilities of tumor cells [28]. Furthermore, the mTOR signaling pathway is also involved in regulating the cell’s response to external stimuli. In breast cancer cells, treatment strategies targeting mTOR have been found to effectively inhibit the EMT process, thereby improving the effectiveness of chemotherapy [29]. In pancreatic cancer, mTOR affects cell proliferation and metastatic ability by regulating the phosphorylation of YBX1, further promoting the occurrence of EMT [30]. Therefore, by modulating the mTOR signaling pathway, it is possible to effectively intervene in the changes in the extracellular matrix, thereby affecting the invasiveness and metastatic ability of tumors.

In this study, we discovered that BB inhibited the activation of the PI3K/AKT/mTOR signaling pathway both in vitro and in vivo. Then, we examined the effect of BB-containing serum on the EMT in PLGC cells. During the occurrence of EMT, the expression level of E-cadherin, a calcium-dependent adhesion molecule associated with EMT, decreases, whereas the expression level of N-cadherin, another type of cadherin molecule, increases [31]. E-cadherin is a transmembrane glycoprotein primarily expressed in epithelial cells and plays an essential role in maintaining cell-to-cell adhesion [32]. N-cadherin is predominantly expressed in the interstitial tissue, and its upregulated expression serves as a biomarker for EMT, which confers enhanced migratory and invasive capabilities on cells [33]. This study demonstrated that BB-containing serum causes an upregulation of E-cadherin expression and a downregulation of N-cadherin expression in PLGC cells. These findings suggest that BB may inhibit the EMT of gastric mucosal epithelial cells by modulating the PI3K/AKT/mTOR signaling pathway, thereby reversing the progression of PL-CAG. Although our current study primarily focused on the expression changes of E-cadherin and N-cadherin, we acknowledge the necessity of evaluating other EMT markers to comprehensively understand the underlying mechanisms. In future studies, we plan to systematically investigate additional markers, including vimentin and Snail, thereby providing more robust evidence for BB’s inhibitory effects on EMT.

This study has preliminarily explored the mechanism by which BB inhibits the EMT of gastric mucosal epithelial cells by suppressing the PI3K/AKT/mTOR signaling pathway, thereby potentially reversing the PL-CAG.

Although we have endeavored to ensure the scientific rigor of the study design and the reliability of the results, several limitations of this research warrant acknowledgment. First, the potential pharmacological relevance between active compounds and core targets necessitates further investigation. Second, although in vitro experiments and rat models offer valuable initial insights into drug mechanisms, they cannot fully simulate the intricate physiological and pathological conditions of humans. Consequently, this may result in discrepancies between the drug effects observed in these models and those encountered in clinical settings. Third, our study predominantly examined the inhibitory effects of BB on the PI3K/AKT signaling pathway but did not explore activators of this pathway in depth. As a result, our comprehension of the regulatory mechanisms governing this pathway remains limited, which hinders a thorough elucidation of BB’s complete pharmacological profile. Fourth, while the BB extract comprises multiple compounds, we did not conduct quantitative analysis of specific components. This limitation restricts our capacity to accurately pinpoint the primary compounds responsible for the observed effects or to exclude potential interference from other constituents.

In future research, we aim to compare the binding energy of components with their respective targets against that of known target inhibitors, thereby assessing pharmacological relevance. Additionally, we are actively planning collaborations with clinical research institutions to carry out pharmacokinetic and toxicological studies, which will provide more comprehensive data support for subsequent clinical trials. Activators of the PI3K/AKT signaling pathway will be introduced to investigate BB’s regulatory effects on this pathway more comprehensively via both activation and inhibition approaches. In vitro cell experiments and animal models will be employed to screen specific compounds with significant therapeutic efficacy, followed by an in-depth investigation into their mechanisms of action. Furthermore, human trials and combination therapy studies can be conducted to validate the safety and efficacy of BB in humans, thereby providing robust evidence for its clinical application. Through combination therapy research, the practical feasibility and advantages of BB in real-world clinical settings will be evaluated, aiming to enhance therapeutic outcomes while minimizing side effects.

## 4. Materials and Methods

### 4.1. Drugs and Reagents

The BB was obtained from Hebei Meiwei Pharmaceutical Co. (Anguo, China), and methanol, acetonitrile, and formic acid were sourced from Thermo Fisher Scientific (Waltham, MA, USA). N-methyl-N’-nitro-N-nitrosoguanidine (MNNG) and sodium salicylate were obtained from Aladdin. Vitac tablets were acquired from Beihai Sunshine Pharmaceutical Co. Rat GAS, PGI, PGII, IL-6, TNF-α, and IL-1β ELISA kits were sourced from Shanghai Thrive Color Biotechnology Co. The 1640 medium, fetal bovine serum, 0.25% trypsin-EDTA, and PBS were obtained from Gibco (New York, CA, USA). CCK-8 reagents were acquired from Abbkine. The serum-free freezing solution was obtained from Suzhou Xinsaimei Biotechnology Co. (Suzhou, China). GAPDH, β-Tubulin, E-Cadherin, and N-Cadherin antibodies were acquired from Wuhan Proteintech Co. (Wuhan, China). IL-6, IL-1β, and TNF-α antibodies were obtained from SANTA CRUZ BIOTECHNOLOGY, INC. Akt, p-Akt, PI3K, and p-PI3K antibodies were sourced from Affinity. mTOR and p-mTOR antibodies were acquired from ZenBio (Chengdu, China). The HRP-goat anti-rabbit antibody was purchased from Cohesion, and the HRP-goat anti-mouse antibody was obtained from Tianjin Simu Biotechnology Co. (Tianjin, China).

### 4.2. Preparation of BB

BB was crushed, passed through an 800-mesh sieve, and subjected to reflux extraction. A total of 150 g of the sample was weighed into a round-bottomed flask, soaked immersed in water equivalent to five times its weight in water for 30 min, and subsequently extracted by heated reflux three times, each for 2 h. The filtrate was filtered, combined, and centrifuged at 3000 r/min for 10 min to remove the residue. The supernatant was then concentrated under vacuum to a concentration of 2 g/mL of raw drug and stored in a −80 °C refrigerator. In in vitro experiments, drug-containing serum was utilized. The preparation procedure for the drug-containing serum was as follows: Rats were orally administered the water extract of BB for three consecutive days. Subsequently, blood was collected from the abdominal aorta and placed into a coagulant tube. The samples were kept on ice for 30 min, then centrifuged at 3000 rpm for 15 min, and finally stored at −80 °C for subsequent use. Under the same conditions, the rat blood was placed in the anticoagulant tube and the above operation was repeated to obtain the drug-containing plasma of BB.

### 4.3. Chromatographic and Mass Spectrometric Conditions

The UPLC-QE-Orbitrap-MS/MS was utilized to analyze samples from both the blank plasma group and the drug-containing plasma group of BB. The data collected were then imported into the UNIFI platform for rapid matching and compared against the confidential retention times and secondary mass spectrometry fragment ion information for each component in the chemical composition analysis of BB. Chromatographic conditions: the chromatographic column was ACQUITY UPLC HSS T3 (100 mm × 2.1 mm, 1.8 μm), the column temperature was set at 45 °C, and the gradient elution was carried out using A (0.1% formic acid-water) -B (acetonitrile) as the mobile phase. The elution program was as follows: 0–2 min, 95–95%A; 2–4 min, 95–70%A; 4–8 min. 70–50%A; 8–10 min, 50–20%A; 10–14 min, 20–0%A; 14–15 min, 0–0%A; 15–15.1 min, 0–95%A; 15.1–16 min, 95– 95%A; the flow rate was 0.35 mL/min, and the injection volume was 5 μL. Mass spectrometry (MS) conditions: electrospray ion source (ESI source), TurboV ion source, source temperature of 550.0 °C. The source injection voltage was −4500.0 V. The atomization gas (gas1) and heating gas (gas2) were both 55.0 psi, the air curtain gas was 25.0 psi, and the negative ions were monitored by the multi-reaction monitoring (MRM) mode.

### 4.4. Animals and Model Establishment

Specific Pathogen Free (SPF)-grade 5-week-old Wistar male rats (200 ± 20 g), provided by Specific Pathogen Free (Beijing) Biotechnology Co. (Beijing, China). The relevant experiments were performed after one week of adaptive rearing in a constant temperature and humidity environment with a temperature of 22 °C and a humidity of 50%. Animal experiment operation license number: SCXK (Ji) 2022-010. The experimental procedures involving the animals adhered to the National Institutes of Health Guide for the Care and Use of Laboratory Animals and were granted approval by the Animal Care and Use Committee of Medical Ethics of the Hebei University of Chinese Medicine (approval number DWLL202304006). In total, 15 rats were randomly selected from 100 Wistar male rats as the control (Ctrl) group, and the remaining rats were modeled by a three-factor method with reference to the literature [34]: (i) MNNG was prepared as an aqueous solution of 180 μg/mL protected from light for free drinking, and was changed daily; (ii) starvation and satiety disorders: 2 days were for full feeding and 1 day was for fasting; (iii) after fasting, 0.5 mL of 2% sodium salicylate was administered orally to rats at a concentration of 0.5 mL/100 g. Starting from the 12th week of modeling, blood was taken from the inner canthus of the eye every two weeks, and the serum expression levels of GAS, PGI, PGII, IL-6, IL-1β, and TNF-α were detected in each group of rats using ELISA kits (Zhuocai Biotechnology Co., Shanghai, China). After the differences in serum indexes appeared, two rats in the modeling group were randomly selected and killed every two weeks. The histopathological damage of the stomach in each group was observed by HE staining until both rats showed a reduction in the number of intrinsic glands atrophy or disappearance in the gastric mucosal epithelial cells, which was regarded as the success of the modeling. The general condition of the rats was observed during the period and recorded by weekly weighing at regular intervals. The treatment was started on the next day after successful modeling. The administered dose was converted according to the equivalent human clinical dose and the rat body surface area coefficient [35]: 0.32 g/kg for the Vitac group, 0.63 g/kg, 1.26 g/kg and 2.52 g/kg for the BB low-dose group (BB-L), BB medium-dose group (BB-M), and BB high-dose group (BB-H), respectively, and was administered once a day for 12 weeks, while the Ctrl group and the Model group were given a normal diet every day. The rats were euthanized under anesthesia at the end of the experiment after a 12 h fast with access to water. The blood was removed from the abdominal aorta and centrifuged at 3000 r/15 min after being placed on ice for 30 min, then the supernatant was stored at −80 °C for measurement. The gastric tissue was dissected along the greater curvature of the stomach to observe the general changes of the gastric mucosa and photographed, part of the gastric tissue was frozen in liquid nitrogen for spare use and the remaining tissue was kept in paraformaldehyde for fixation.

### 4.5. Histological Analysis

Fix the gastric tissue in 10% neutral formalin for a duration of 24 h. Following dehydration, embed the tissue in paraffin wax and section it to a thickness of 5 μm. Dewax using xylene, perform gradient ethanol hydration, and apply hematoxylin and eosin staining. Subsequently, dehydrate with ethanol, clear with xylene, and mount using neutral balsam. Finally, examine the histological sections under an optical microscope.

### 4.6. Enzyme-Linked Immunosorbent Assay

In accordance with the provided instructions, serum levels of GAS, PGI, PGII, IL-1β, IL-6, and TNF-α were quantified utilizing ELISA kits.

### 4.7. Network Pharmacology Analysis

Taking 42 prototype compounds of blood-entering components in BB as the research objects, the SMILES numbers of the components were entered into the Swiss Target Prediction database (http://swisstargetprediction.ch, accessed on 6 August 2024), and the targets with ‘probability’ values greater than 0 were combined and the duplicates were deleted to obtain the targets of the components. The targets were analyzed in the OMIM (https://www.omim.org, accessed on 7 August 2024), Genecards (https://www.genecards.org, accessed on 7 August 2024), and DisGeNET (https://www.disgenet.org, accessed on 7 August 2024) databases under the names ‘Chronic atrophic gastritis’, ‘atrophic gastritis’, ‘intestinal metaplasia’, ‘dysplasia’, and ‘precancerous lesions of gastric cancer’, which were searched for disease targets. The components were intersected with the disease targets to obtain potential targets for the treatment of precancerous lesions of chronic atrophic gastritis by BB. Potential targets were inputted into the STRING platform (https://string-db.org, accessed on 8 August 2024) for visualization, and core targets were screened according to MCODE using Cytoscape 3.9.1 software. The key targets were imported into the DAVID database (https://davidbioinformatics.nih.gov, accessed on 9 August 2024), limited the study species to human, and subjected to molecular function (MF), biological process (BP) and cellular component (CC) analysis. Additionally, pathway enrichment analysis was performed using KEGG, followed by visualization of the results. using KEGG, and visualization of the results. The composite drug–components–diseases–targets–pathway network was constructed by importing BB, ingredients, potential targets, and pathways into Cytoscape 3.9.1 software for visualization, and the network parameters were analyzed and screened to identify the potential active ingredients of BB that could play a therapeutic role in the treatment of PL-CAG. The potential active ingredients were molecularly docked with the top 11 core targets. The structural formulae of the ligand small molecules were downloaded from the PubChem database (https://pubchem.ncbi.nlm.nih.gov, accessed on 11 August 2024) in SDF format, and the structural formulae of the components were converted to PDB format using Open Babel 3.1.1 and processed for structure optimization. The crystal structures of the receptor proteins were obtained from the RCSB PDB database (https://www.rcsb.org, accessed on 11 August 2024) and operations such as dehydration and hydrogenation were performed by PyMol 1.8.0.0. The receptor protein and ligand small molecules were semiflexible docked in AutoDockTools-1.5.6 to obtain the corresponding binding energies.

### 4.8. Cell Culture

GES-1 cells were cultured in 1640 medium containing 10% fetal bovine serum at 37 °C in a 5% CO_2_ incubator. When the cells grew to 80–90% fusion, 10 μM MNNG was added to induce GES-1 cells to become PLGC model cells [36].

### 4.9. Cell Cloning Assay

Cells from each experimental group were spread in 6-well plates with 200 cells/well, and placed in the incubator for 1 week, during which the liquid was changed normally. The cultured cells were washed twice with PBS, fixed with 4% paraformaldehyde for 15 min, then stained with crystal violet for 15 min, and the residual crystal violet was washed with purified water, then photographed and counted.

### 4.10. Wound Healing Assay

The cells were evenly spread in 6-well plates, the density of which was suitable for overnight fusion of 90–100%. After making scratches with a 200 µL tip, the width of the scratches in the same position was observed under a microscope at 6 h, 12 h, 24 h, and 48 h, and pictures were taken and quantitatively processed by using the software of ImageJ-win64.

### 4.11. Invasion Assays

We put the Transwell into a 24-well plate, diluted Matrigel gel with serum-free 1640 medium at 8:1 in the upper well, discarded the excess liquid in the upper layer after solidification, added 100 μL of serum-free 1640 medium, and continued to incubate for 30 min for basement membrane hydration. The upper well of Transwell after basement membrane hydration was taken out and the liquid was discarded. A suspension containing 2 × 10^3^ cells in 200 μL was added to the upper well, and it was left to stand for 15 min. A total of 500 μL of 1640 medium containing 20% fetal bovine serum was added to the lower well. The 24-well plate was incubated in an incubator at 37 °C, 5% CO_2_ for 48 h. The culture medium was removed from the upper and lower wells and washed twice with PBS, and the matrix glue inside the upper chamber was carefully wiped away with a swab. Then, it was fixed with paraformaldehyde, stained with crystal violet, and photographed for counting.

### 4.12. Western Blot

Total proteins were extracted from tissues and cells. The total protein concentration was determined by BCA method. The protein sample is mixed with 5 × loading buffer at a ratio of 1:4, and denatured at 100 °C for 10 min. The electrophoresis voltages of the polyacrylamide gels were set at S1: 75 V, 35 min, and S2: 110 V, 95 min. The membrane was transferred with 0.45 μm PVDF membrane, closed with 5% skimmed milk powder prepared using 1× TBST for 2 h, incubated with diluted primary antibody at 4 °C overnight, and then washed 3 times with TBST for 10 min each time. After that, the membrane was reacted with horseradish peroxidase-coupled secondary antibody at room temperature for 1 h, washed 3 times with TBST, and ECL was added to develop and expose. The protein bands were quantified using ImageJ-win64, and the relative protein expression was calculated using GAPDH and β-Tubulin as internal references.

### 4.13. Statistical Analysis

Statistical analysis of the data was performed using GraphPad Prism 10.1 software. All the data were represented as mean ± SD. One-way ANOVA was used to compare the data between groups with normal distribution and homogeneity of variance. Pairwise comparison between groups was performed by the least significant difference method (LSD method). If the variance was not uniform, the rank sum test was used. *p* < 0.05 meant the difference was statistically significant, and *p* < 0.01 meant the difference was statistically significant.

## 5. Conclusions

**Figure 8:**
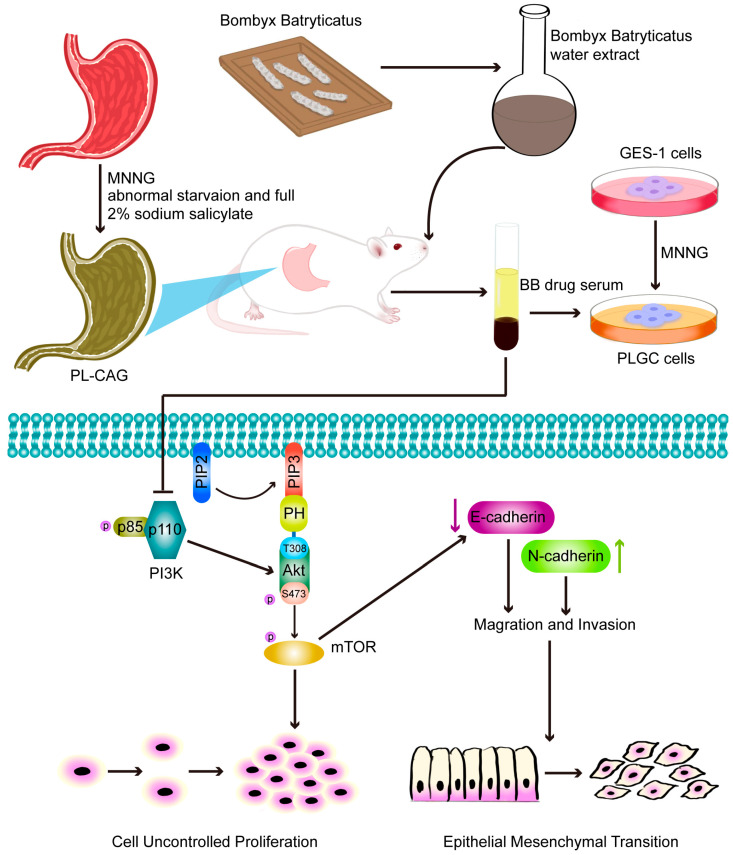
Schematic diagram of the regulatory mechanism of BB in the treatment of CAG. BB can restrain uncontrolled proliferation, migration, and invasion by regulating the PI3K-Akt pathway to inhibit CAG progression.

In conclusion, this study first confirmed through in vivo and in vitro pharmacodynamic experiments that BB can effectively improve the pathological changes and inflammatory progression of PL-CAG. Then, through UPLC-QE-Orbitrap-MS/MS analysis, we identified BB prototype components that can enter the bloodstream. Through network pharmacology analysis, we have identified the effective components, potential core targets, and action pathways of BB treatment for PL-CAG. Mechanism research has revealed that BB effectively inhibits the activation of the PI3K/AKT/mTOR signaling pathway, thereby attenuating the proliferation, migration, and invasion of gastric epithelial cells and reversing the progression from CAG to PLGC. These findings suggest that BB may serve as a promising therapeutic agent for CAG treatment, especially in cases progressing to PLGC (Figure 8).

## Figures and Tables

**Table 1:** Prototype compounds of blood-entering component information of BB.

No.	Name	Formula	RT/min	*m*/*z*	Ion Mode
1	(−)-*N*-methylcrotonosine	C_18_H_19_NO_3_	4.23	320.125	POS
2	(+)-3-Oxo-alpha-ionol	C_13_H_20_O_2_	8.55	253.1446	NEG
3	Rutin	C_27_H_30_O_16_	4.71	611.1612	POS
4	1-[(2R,3R,4S,5S)-3,4-dihydroxy-5-(hydroxymethyl)oxolan-2-yl]pyrimidine-2,4-dione	C_9_H_12_N_2_O_6_	1.22	243.0621	NEG
5	2-[(1R)-4-methyl-1-cyclohex-3-enyl]prop-2-enyl acetate	C_12_H_18_O_2_	9.58	239.129	NEG
6	6-Methyl-7-(3-oxobutyl)bicyclo [4.1.0]heptan-3-one	C_12_H_18_O_2_	7.78	239.129	NEG
7	7,10-Pentadecadiynoic acid	C_15_H_22_O_2_	10.9	235.1691	POS
8	Acanthoside B	C_28_H_36_O_13_	5.31	579.2089	NEG
9	A-dihydroconiferyl ether	C_20_H_26_O_7_	5.05	401.1572	POS
10	Allantoin	C_4_H_6_N_4_O_3_	0.93	157.0369	NEG
11	Allitol	C_6_H_14_O_6_	0.91	181.072	NEG
12	Aspartylphenylalanine	C_13_H_16_N_2_O_5_	3.83	281.1134	POS
13	Astragalin	C_21_H_20_O_11_	5.07	471.0901	POS
14	Azalein	C_22_H_22_O_11_	5.42	463.123	POS
15	Carthamone	C_21_H_20_O_11_	5.07	447.0938	NEG
16	Chinensiolide b	C_15_H_20_O_4_	6.09	263.1289	NEG
17	Cyclo(Phe-Pro)	C_14_H_16_N_2_O_2_	5.12	245.1282	POS
18	Cyclo(Pro-Val)	C_10_H_16_N_2_O_2_	4.54	241.1196	NEG
19	Diisooctyl phthalate	C_24_H_38_O_4_	14.65	391.2838	POS
20	Hexahydropseudoionone	C_13_H_26_O	10.88	243.1964	NEG
21	Hexitol	C_6_H_14_O_6_	0.88	205.0682	POS
22	Hyperoside	C_21_H_20_O_12_	4.82	465.1031	POS
23	Inosine	C_10_H_12_N_4_O_5_	1.37	291.0699	POS
24	Isololiolide	C_11_H_16_O_3_	5.4	197.117	POS
25	Isoquercitrin	C_21_H_20_O_12_	4.87	463.0886	NEG
26	LysoPC(0:0/18:2)	C_26_H_50_NO_7_P	10.49	564.3304	NEG
27	Mansonone G	C_15_H_16_O_3_	9.19	262.1436	POS
28	*N*-amidinoproline	C_6_H_11_N_3_O_2_	1.22	158.0924	POS
29	Nepitrin	C_22_H_22_O_12_	5.07	477.1042	NEG
30	Nigakilactone f	C_22_H_32_O_7_	6.08	453.2132	NEG
31	Nigakilactone m	C_21_H_30_O_7_	5.7	439.1975	NEG
32	Pantothensaure	C_9_H_17_NO_5_	2.83	220.1179	POS
33	Pedaliin	C_22_H_2_2O_12_	5.05	479.1181	POS
34	Perilloside A	C_16_H_26_O_6_	5.8	359.1726	NEG
35	Pratensein 7-*O*-glucopyranoside	C_22_H_22_O_11_	5.44	461.1093	NEG
36	Quercetin 3-*O*-neohesperidoside	C_27_H_30_O_16_	4.7	609.1467	NEG
37	Riboflavin	C_17_H_20_N_4_O_6_	4.4	377.1457	POS
38	(+)-methyl selina-4,11-dien-14-oate	C_16_H_24_O_2_	10.96	247.1703	NEG
39	Secoisolariciresinol monoglucoside	C_26_H_36_O_11_	4.97	525.2309	POS
40	Senkyunolide-F	C_12_H_14_O_3_	8.08	251.0925	NEG
41	Succinyladenosine	C_14_H_17_N_5_O_8_	3.05	384.1148	POS
42	Torachrysone-8-*O*-beta-D-glucoside	C_20_H_24_O_9_	5.98	389.1235	NEG

## Data Availability

The original contributions presented in the study are included in the article, further inquiries can be directed to the corresponding authors.

## Abbreviations

BB	Bombyx Batryticatus
CAG	chronic atrophic gastritis
PL-CAG	chronic atrophic gastritis with precancerous lesions
UPLC-QE-Orbitrap-MS/MS	Ultra performance liquid chromatography-quadrupole-electrostatic field orbital trap high-resolution mass spectrometry
IM	Intestinal metaplasia
Dys	Dysplasia
PLGC	Precancerous lesions of gastric cancer
TCM	Traditional Chinese medicine
GC	Gastric cancer
PPI	Protein–protein interaction
GO	Gene ontology
KEGG	Kyoto Encyclopedia of Genes Genomics
MCODE	Molecular Complex Detection
TIC	Total ion chromatograms
EMT	Epithelial–mesenchymal transition
